# Phenylethynyl-Terminated Imide Oligomer-Based Thermoset Resins

**DOI:** 10.3390/polym16202947

**Published:** 2024-10-21

**Authors:** Minju Kim, Kiyeong Kim, Joon Hyuk Lee, Eunkyung Jeon, Jungkun Song, Jaeho Choi, Hyeonuk Yeo, Ki-Ho Nam

**Affiliations:** 1 Department of Textile System Engineering, Kyungpook National University, Daegu 41566, Republic of Korea; 2 Agency for Defense Development, Yuseong P.O. Box 35, Daejeon 34186, Republic of Korea; 3 Department of Chemistry Education, Kyungpook National University, Daegu 41566, Republic of Korea; 4 School of Applied Chemical Engineering, Kyungpook National University, Daegu 41566, Republic of Korea

**Keywords:** thermoset resin, phenylethynyl-terminated imide oligomer, structure parameters, composite material matrix

## Abstract

Phenylethynyl-terminated imide (PETI) oligomers are highly valued for their diverse applications in films, moldings, adhesives, and composite material matrices. PETIs can be synthesized at varying molecular weights, enabling the fine-tuning of their properties to meet specific application requirements. Upon thermal curing, these oligomers form super-rigid network structures that enhance solvent resistance, increase glass-transition temperatures, and improve elastic moduli. Their low molecular weights and melt viscosities further facilitate processing, making them particularly suitable for composites and adhesive bonding. This review examines recent advancements in developing ultra-high-temperature PETIs, focusing on their structure–processing–properties relationships. It begins with an overview of the historical background and key physicochemical characteristics of PETIs, followed by a detailed discussion of PETIs synthesized from monomers featuring noncoplanar configurations (including kink and cardo structures), fluorinated groups, flexible linkages, and liquid crystalline mesogenic structures. The review concludes by addressing current challenges in this research field and exploring potential future directions.

## 1. Introduction

Thermosetting polyimides (PIs) based on phenylethynyl-terminated imide (PETI) oligomers are among the most critical high-performance resin matrices for high-temperature aerospace and aircraft applications [1,2,3]. Upon curing, PI resins form highly rigid network structures through the additional reaction of phenylethynyl groups, imparting exceptional thermo-oxidative stability, mechanical strength, and resistance to heat and radiation [4,5,6].

PETIs are commonly synthesized through a conventional two-step procedure and subsequently converted into thermosetting polyimides through thermal curing at approximately 370 °C for 1–2 h [3,7,8]. The synthesis involves the reaction of a dianhydride, diamine monomers, and 4-phenylethynyl phthalic anhydride (PEPA) as an end-capping agent. Consequently, the chemical structures of the dianhydride and diamine monomers, along with the molecular weight of the PETIs, can be adjusted to tailor their properties, enabling the optimization of performance for specific applications [3,9,10,11,12,13]. Due to their exceptional properties, PETIs with diverse structures have been widely studied.

The PETI-5, derived from 3,3′,4,4′-biphenyltetracarboxylic dianhydride (s-BPDA) and developed at NASA Langley Research Center (LaRC), with a targeted molecular weight of 5000 g mol^−1^, demonstrates an excellent combination of high fracture toughness, heat resistance, and processability [7]. It exhibits a minimum melt viscosity of 1000 Pa∙s at 371 °C and a cured glass transition temperature (*T*_g_) of 270 °C. Studies by Scola et al. revealed that substituting s-BPDA units with 4,4′-(hexafluoroisopropylidene)-diphthalic anhydride (6FDA) units significantly reduces the melt viscosity with minimal impact on *T*_g_ [14]. The 6FDA-based PETI exhibited a minimum melt viscosity of 48 Pa∙s at 337 °C and a cured *T*_g_ of 268 °C [15,16]. Around the year 2000, Connell et al. [17] introduced PETI-298, PETI-330, and PETI-375, synthesized using symmetric or unsymmetric biphenyl dianhydride. These resins exhibited a distinct *T*_g_ of 298 °C, 330 °C, and 375 °C, respectively, making them suitable for applications across various service temperatures. Each resin demonstrated low viscosity and excellent melt stability at 280 °C, facilitating resin transfer molding (RTM) processing into composites with robust mechanical properties [18,19]. Recently, the use of unsymmetric dianhydrides in RTM resin development has increased. Substituting symmetric BPDA units with asymmetric BPDA units enhanced the cured *T*_g_ and improved the processability of PI resins [20,21]. The asymmetric structure of a-BPDA needs more space for molecular motion, requiring more energy, which leads to an increase in the glass transition temperature (*T*_g_). At the same time, this asymmetric and non-planar structure hinder close chain packing, thereby improving processability [8,20,21]. Further advancements include developing PI resins with low melting temperatures using 2,3,3′,4′-oxydiphthalic anhydride (a-ODPA) and 2,3,3′,4′-benzophenone anhydride (a-BTDA) as precursors, with phenylethynyl groups as terminal moieties [8,22,23]. This innovation has significantly advanced research in this field.

The ongoing development of PETI resins with high *T*_g_ and low melt viscosities remains a critical focus area. These resins are preferred for their ability to combine desirable properties, including exceptional fracture toughness and resistance to microcracking. This review summarizes recent advances in PETI resins, emphasizing molecular design and the relationships between structure–processing–properties. The insights provided aim to pave the way for further research into high-temperature composite resins, contributing to advancements in the field.

## 2. PETI Resins with Intrinsic Structure

### 2.1. Isomeric Structure

**Scheme 1:**
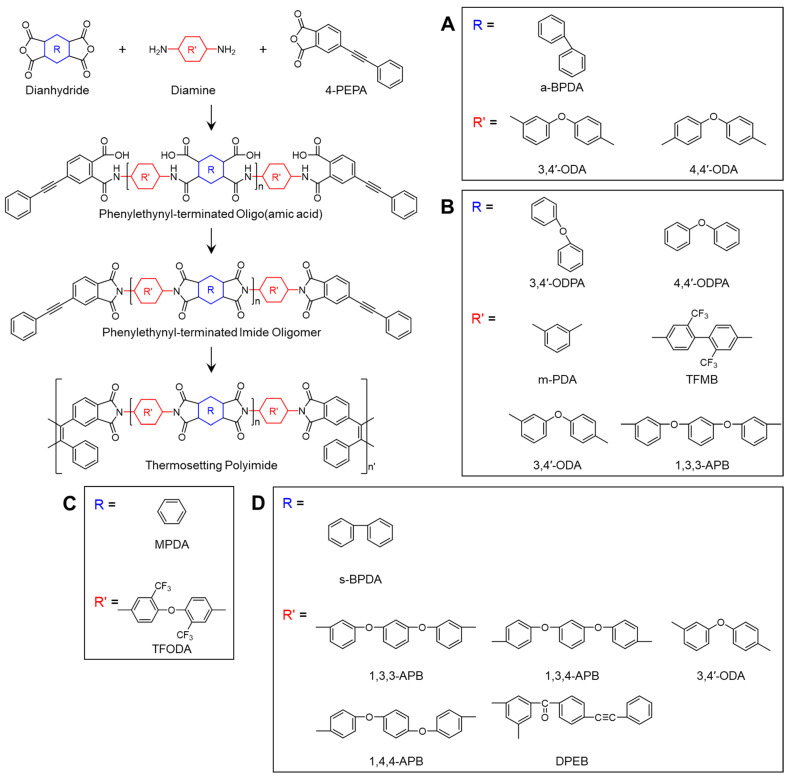
Chemical structures of PETIs with isomeric configurations: (**A**) Oligo-n [3]; (**B**) PI-n [8]; (**C**) Oligo-n [33]; (**D**) Pn and PETI-RTM [34].

Isomers are molecules that share the same chemical formula but exhibit different properties due to variations in the arrangement of atoms or their spatial orientation, and they are classified into two main types: structural isomers and stereoisomers. Structural isomers differ in their bonding and arrangement of atoms, while stereoisomers differ in the spatial orientation of their atoms. Consequently, isomers with the same molecular formula can possess distinct physical and chemical properties. These characteristics have led to extensive studies on polyimides synthesized using isomeric dianhydrides or diamines [24,25,26,27]. Generally, using isomeric monomers results in a less ordered and more flexible three-dimensional structure [28], enabling the fabrication of processable polyimides without significant loss of thermal stability [27]. For instance, a PETI based on 2,3,3′,4′-biphenyltetracarboxylic dianhydride (a-BPDA) exhibits a higher *T*_g_ than one based on 3,3′,4,4′-biphenyltetracarboxylic dianhydride (s-BPDA). This difference is attributed to the asymmetric structure of a-BPDA, which suppresses internal rotation around the biphenyl linkage compared to s-BPDA. The a-BPDA structure requires a larger sweep volume for crankshaft motion, necessitating more energy (i.e., higher temperature), thereby resulting in a higher *T*_g_. Conversely, isomer-based PETIs such as a-BPDA exhibit enhanced processability. For example, TriA-PI, a PETI based on a-BPDA, exhibited improved solubility and reduced viscosity compared to a PETI based on s-BPDA due to the asymmetric and non-planar structure of a-BPDA [3,20]. The characteristic charge transfer complex (CTC) in imides is formed through π-electron conjugation in polyimides, where the nitrogen atom acts as the electron donor and the carboxyl group of the dianhydride functions as the electron acceptor [29,30]. This results in strong inter- and intrachain interactions [31]. Therefore, the introduction of isomeric structures into the molecular backbone can disrupt the chain packing due to the asymmetric nature of the isomers, reducing the CTC formation and molecular interactions, which ultimately enhance processability [32]. This section discusses PETIs derived from isomeric monomers. The chemical structures used in the studies presented in this section are shown in Scheme 1.

**Figure 1:**
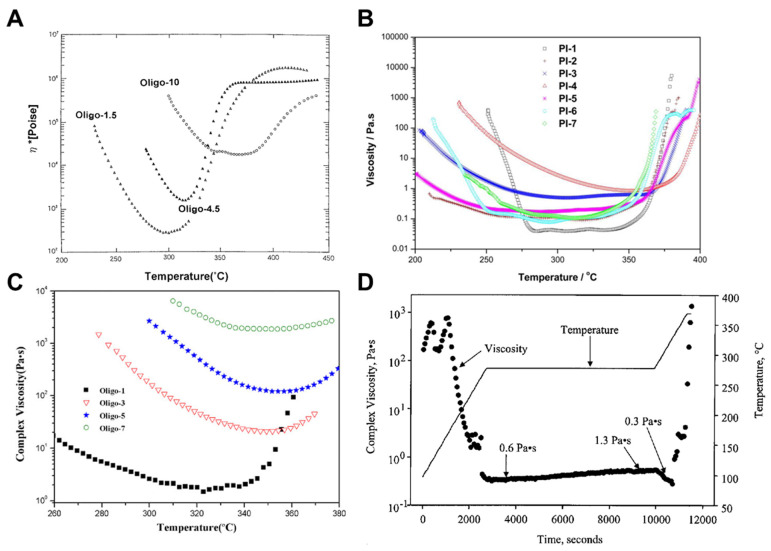
Complex viscosity curves: (**A**) Oligo-n (reprinted with permission from [3], Copyright 2001, SAGE Publications); (**B**) PI-n (reprinted with permission from [8], Copyright 2015, SAGE Publications); (**C**) Oligo-n (reprinted with permission from [33], Copyright 2018, Wiley); (**D**) P4 (reprinted with permission from [34], Copyright 2002, SAGE Publications).

**Figure 2:**
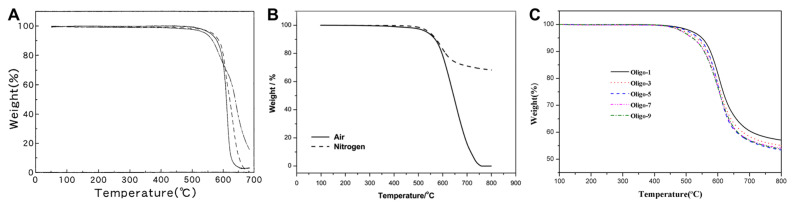
TGA curves: (**A**) The fully cured Oligo-n and polyimide in air; Oligo-1.5 (— · —), Oligo-10 (– – –), PI (a-BPDA/3,4′-ODA; 4,4′-ODA) (50:50) (——) (reprinted with permission from [3], Copyright 2001, SAGE Publications); (**B**) Cured PI-6 in air and in nitrogen (reprinted with permission from [8], Copyright 2015, SAGE Publications); (**C**) The fully cured Oligo-n films in nitrogen (reprinted with permission from [33], Copyright 2018, Wiley).

Yokota et al. [3] reported the development of Triple-A PI (Tri-A PI), an amorphous, asymmetric, addition-type PETI based on a-BPDA, 3,4′-ODA, and 4,4′-ODA. Specifically, Oligo-1.5 (a-BPDA/3,4′-ODA;4,4′-ODA (50:50)/PEPA, n = 1.5) demonstrated high solubility in DMAc and exhibited an improved dynamic melt viscosity of 200 Poise at 300 °C. Oligo-10 (n = 10) also showed high solubility in DMAc but exhibited a dynamic melt viscosity of 20,000 Poise at 365 °C. The complex viscosity curves are shown in Figure 1A. Furthermore, while PETI-5 based on s-BPDA exhibited a *T*_g_ of 270 °C, the a-BPDA-based Oligo-1.5 and Oligo-10 exhibited higher *T*_g_ values of 341 °C and 308 °C, respectively, after curing at 370 °C for 1 h. The thermal properties of PETI oligomers and cured resins are summarized in Table 1. Also, the TGA curve in air atmosphere can be seen in Figure 2A.

Wang et al. [8] reported the synthesis and characterization of isomeric PETIs derived from 3,4′-ODPA, an isomer of 4,4′-ODPA. PI-6 (3,4′-ODPA/TFMB;1,3,3-APB (50:50)/PEPA) exhibited a minimum melt viscosity of 0.08 Pa·s at 295 °C, whereas PI-7 (4,4′-ODPA/TFMB;1,3,3-APB (50:50)/PEPA) demonstrated a minimum melt viscosity of 0.11 Pa·s at 312 °C. This difference was attributed to the asymmetric structure of 3,4′-ODPA, which reduces chain linearity and intermolecular bonding. PI-6 maintained a viscosity below 1 Pa·s even after being held at 250 °C for 2 h, indicating its suitability for RTM processing at this temperature, whereas PI-7 exhibited higher viscosity at 250 °C, making it unsuitable for injection molding. The complex viscosity curves are shown in Figure 1B. The *T*_g_ of the cured PI-6 and PI-7, measured by DSC and DMA, were similar, at 304 °C and 292 °C, respectively, and 352 °C and 356 °C. Both cured PI thermosets demonstrated high thermal stability, with a 5% weight loss temperature exceeding 540 °C. The TGA curves are shown in Figure 2B. After curing, PI-6 exhibited a tensile strength of 45 MPa and an elongation of 3.38%, while PI-7 showed a tensile strength of 66 MPa and an elongation of 1.86%, suggesting that the 4,4′-ODPA-based cured resins had higher stiffness. The thermal properties of PETI oligomers and cured resins are summarized in Table 1.

Fang et al. [33] were the first to report the development of a PETI based on MPDA, an isomer of PMDA. Oligo-5 (MPDA/TFODA/PEPA, n = 5) was completely soluble in both high-boiling-point solvents such as NMP and low-boiling-point solvents, including THF, 1,4-dioxane, and acetone, whereas Oligo-PMDA (PMDA/TFODA/PEPA, n = 5) was insoluble in low-boiling-point solvents. Furthermore, Oligo-5 exhibited a minimum complex melt viscosity of 120 Pa·s at 354 °C, while Oligo-PMDA did not melt. These characteristics were attributed to the bent structure of MPDA, which suppresses molecular aggregation and increases free volume. The complex viscosity curves are shown in Figure 1C. After curing, Oligo-5 demonstrated excellent thermal stability with a high *T*_g_ of 373 °C and a *T*_d5%_ of 539 °C, attributed to the rigidity of MPDA. These results are presented in Table 1 and Figure 2C. It also exhibited outstanding mechanical properties, including a tensile strength of 80 MPa, a tensile modulus of 1.9 GPa, and an elongation of 7.1%.

Smith et al. [34], at NASA Langley Research Center, reported PETIs synthesized using s-BPDA and the isomeric diamines 1,3,3-APB, 1,3,4-APB, and 1,4,4-APB. Before curing, the *T*_g_ of PETI-RTM (s-BPDA/1,3,3-APB; 3,4′-ODA (75:25)/PEPA) and P4 (s-BPDA/1,3,4-APB; 3,4′-ODA (75:25)) were 132 °C and 139 °C, respectively. After curing, the *T*_g_ values increased to 258 °C for PETI-RTM and 298 °C for P4, while P5 (s-BPDA/1,4,4-APB; 3,4′-ODA (75:25)/PEPA) did not exhibit a *T*_g_ in the DSC analysis. These results suggest that *T*_g_ increases with the rigidity of the APB isomer (1,4,4-APB > 1,3,4-APB > 1,3,3-APB). The thermal properties of PETI oligomers and cured resins are summarized in Table 1. The melt viscosities of PETIs based on 1,3,3-APB and 1,3,4-APB showed no significant difference; however, the melt viscosity of P5, based on 1,4,4-APB, was five times higher than that of P4, based on 1,3,4-APB. The complex viscosity curves are shown in Figure 1D.

### 2.2. Noncoplanar Structures (Kink, Spiro, and Cardo Structures)

#### 2.2.1. Kink Structure

**Scheme 2:**
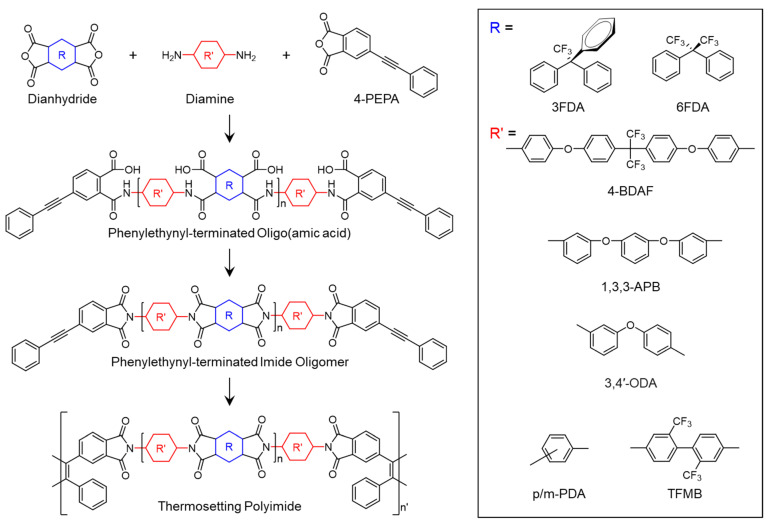
Chemical structures of PETIs with kinked configurations: PETI, PE-3F, and PE-6F oligoimides [14].

The kink structure can be described as a crank and twisted non-planar configuration [35], which distorts polymer chains, reducing packing density and increasing chain flexibility [36]. This distortion partially hinders the formation of charge transfer complexes (CTCs) within or between molecules, allowing for the formation of colorless polyimide films with high transparency and improved solubility [37]. Similar strategies involving kink structures in PETIs have been explored. The chemical structures used in the study presented in this section are shown in Scheme 2.

**Figure 3:**
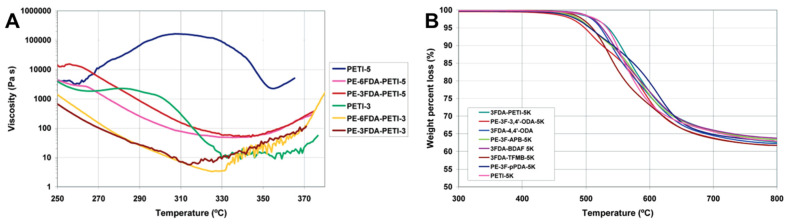
(**A**) Viscosity curves of PE-3F oligoimides; (**B**) TGA curves of PE-3F oligoimides in nitrogen (reprinted with permission from [14], Copyright 2003, American Chemical Society).

Scola et al. [14] reported PETIs featuring kink structures based on 3FDA. PE-3F-PETI-5K (3FDA/3,4′-ODA; APB (85:15)/PEPA) and PETI-5K (s-BPDA/3,4′-ODA; APB (85:15)/PEPA) exhibited *T*_g_ of 218 °C and 225 °C, respectively. Upon curing, the *T*_g_ values increased to 272 °C and 270 °C, which are not significantly different from those of the uncured PETIs. However, while PE-3F-PETI-5K did not display a melt temperature (*T*_m_), PETI-5K showed a *T*_m_ of 349 °C, suggesting that the PETI based on 3FDA possesses a less ordered structure with greater molecular freedom and weaker intermolecular interactions, resulting in an amorphous structure. The thermal properties of PETI oligomers and cured resins are summarized in Table 2. The calculated dihedral angles at the 1,1′ positions of 3FDA and s-BPDA were 84.7° and 42.6°, respectively, indicating a larger degree of out-of-plane twisting for 3FDA and leading to greater intermolecular distances in PE-3F-PETI-5K. At 310 °C, PETI-5K exhibited an initial viscosity of 20,759 Pa·s, which increased to 412,205 Pa·s after 60 min of isothermal treatment at the same temperature. By contrast, PE-3F-PETI-5K showed a significantly lower initial viscosity of 321 Pa·s, rising only to 527 Pa·s after identical treatment, indicating much higher melt stability. The complex viscosity curves are shown in Figure 3A. The difference in melt stability is associated with the electronic environment around the phenylethynyl end groups. The curing reaction of phenylethynyl occurs through thermally induced free radical initiation and propagation. Since radicals can be destabilized by the inductive effect of electron-withdrawing groups, these groups hinder the initiation step of ethynyl bond cleavage. Consequently, at 310 °C, the 3FDA and 6FDA units significantly slow down the crosslinking reaction due to the suppression of initial bond cleavage and radical formation. As a result, PE-3F-PETI-5K exhibits higher melt stability, with a reduced crosslinking rate at the minimum melt viscosity temperature, due to the stabilization of the phenylethynyl end groups. Additionally, the TGA curves in nitrogen atmosphere are presented in Figure 3B.

#### 2.2.2. Cardo Structure

**Scheme 3:**
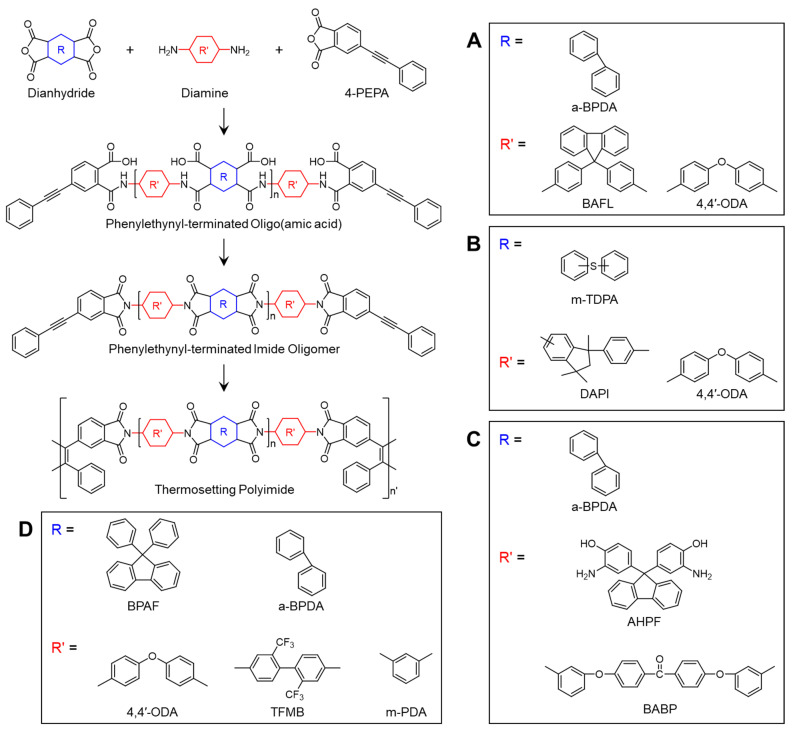
Chemical structures of PETIs with cardo configurations: (**A**) *o*-BAFL-n [44]; (**B**) Oligo-n [45]; (**C**) PI oligomer/Cardo-HPI blends [43]; (**D**) B-O-n, F-O-n, B-T-n, F-T-n, B-P-n, and F-P-n [9].

The term “cardo”, derived from Latin meaning hinge or ring, refers to a structure in which a cyclic unit is integrated into the main polymer chain [35,38]. This structure is similar to a spiro configuration; however, unlike spiro, the cardo structure features only one ring connected to the main chain through a bridging unit or central group. By contrast, the spiro structure consists of two rings that are connected orthogonally through a single common atom [35]. Due to its twisted configuration, the cardo structure hinders chain rotation and loosens chain packing, leading to increased free volume [39,40,41]. Introducing a cardo group into the polymer backbone is a proven method for enhancing solubility [42]. Additionally, the rigid nature of the cardo structure contributes to a high *T*_g_ and excellent thermal stability [43]. This study presents PETIs with cardo structures. The chemical structures used in the studies presented in this section are shown in Scheme 3.

**Figure 4:**
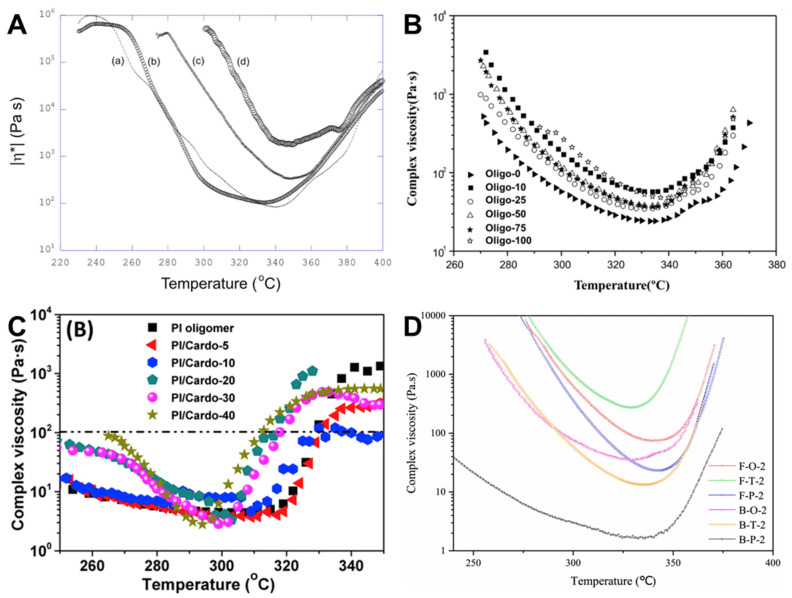
Complex viscosity curves: (**A**) TriA-PI and *o*-BAFL series; ((**a**) TriA-PI (*o*-BAFL-0), (**b**) *o*-BAFL-10, (**c**) *o*-BAFL-25, (**d**) *o*-BAFL-50) (reprinted with permission from [44], Copyright 2006, SAGE Publications); (**B**) Oligo-n (reprinted with permission from [45], Copyright 2018, Springer); (**C**) PI oligomer/Cardo-HPI blends (reprinted with permission from [43], Copyright 2019, Elsevier); (**D**) F-O-2, F-T-2, F-P-2, B-O-2, B-T-2, and B-P-2 (reprinted with permission from [9], Copyright 2023, Elsevier).

Yokota et al. [44] reported on PETIs based on BAFL, incorporating fluorenylidene groups with bulky and rigid cardo structures. The PETI designated as *o*-BAFL-0 (Tri-A) (a-BPDA/4,4′-ODA/PEPA) exhibited 20 wt.% solubility in NMP, with gelation occurring at a 30 wt.% NMP solution. By contrast, *o*-BAFL-50 (a-BPDA/BAFL; 4,4′-ODA (50:50)/PEPA) demonstrated enhanced solubility, showing 40 wt.% solubility in NMP at room temperature and maintaining a homogeneous solution even after two months in a 30 wt.% NMP solution, attributed to the introduction of BAFL. The *T*_g_ values and minimum melt viscosity of the uncured PETIs increased with the content of the rigid BAFL. *o*-BAFL-0 exhibited a *T*_g_ of 218 °C and a minimum melt viscosity of 81 Pa·s at 344 °C, while *o*-BAFL-50 had a *T*_g_ of 273 °C and a minimum melt viscosity of 1810 Pa·s at 349 °C. The complex viscosity curves are shown in Figure 4A. After curing, *o*-BAFL-0 showed a *T*_g_ of 340 °C and a *T*_d5%_ of 556 °C under argon, whereas *o*-BAFL-50 exhibited a *T*_g_ of 362 °C and a *T*_d5%_ of 561 °C. Thermal properties of PETI oligomers and cured resins are summarized in Table 3. The mechanical properties of the cured resin films revealed that *o*-BAFL-0 and *o*-BAFL-50 had tensile moduli of 2.55 GPa and 2.65 GPa, strengths at break of 118 MPa and 112 MPa, and elongations at break of 15.5% and 6.9%, respectively. These trends were attributed to the increased rigidity of the fluorene groups.

**Figure 5:**
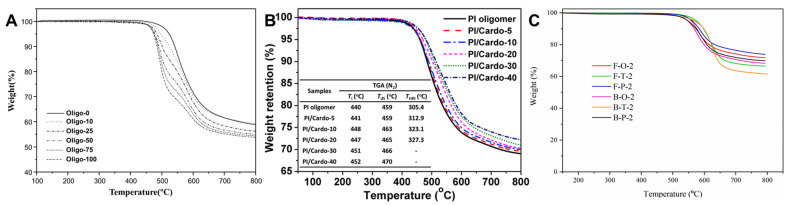
TGA curves: (**A**) Oligo series in nitrogen (reprinted with permission from [45], Copyright 2018, Springer); (**B**) Cured PI/Cardo-HPI thermosets in nitrogen (reprinted with permission from [43], Copyright 2019, Elsevier); (**C**) Thermosetting polyimides in nitrogen (reprinted with permission from [9], Copyright 2023, Elsevier).

Fang et al. [45] introduced PETIs incorporating DAPI, a rigid structure featuring four side groups: three methyl groups and one phenyl group, compared to BAFL, which contains two phenyl groups. Oligo-0 (m-TDPA/4,4′-ODA/PEPA) was highly soluble in high-boiling-point solvents such as DMAc and NMP but only partially soluble in low-boiling-point solvents like THF, CHCl_3_, and 1,4-dioxane. By contrast, Oligo-25 (m-TDPA/DAPI; 4,4′-ODA (25:75)/PEPA) was soluble in both high- and low-boiling-point solvents, attributed to the asymmetric and non-planar structure of DAPI. The minimum melt viscosity of Oligo-0 was 24.1 Pa·s at 334 °C, whereas Oligo-25 exhibited a higher viscosity of 34.7 Pa·s at 331 °C. Despite this, both minimum melt viscosities were below 60 Pa·s around 330 °C, indicating the difference was insignificant. The complex viscosity curves are shown in Figure 4B. The *T*_g_ value of Oligo-0 was 190 °C, whereas Oligo-25 showed an increased *T*_g_ of 205 °C, reflecting the rise in DAPI content. The cured resins of Oligo-0 and Oligo-25 exhibited *T*_g_ of 304 °C and 322 °C, respectively. The thermal properties of PETI oligomers and cured resins are summarized in Table 3. However, their *T*_d5%_ values were 523 °C and 497 °C, indicating a trend of decreased thermal stability with increased DAPI content due to the degradation of aliphatic rings. The TGA curves are shown in Figure 5A. In terms of mechanical properties, Oligo-0 demonstrated a modulus of 1.8 GPa, strength of 90 MPa, and elongation at break of 10.7%, while Oligo-25 exhibited a higher modulus of 2.2 GPa but lower strength of 69 MPa and elongation of 4.8%.

Zhang et al. [43] reported the development of new crosslinked polyimide thermosets by blending BisAHPF-based 9,9-bis(3-(3-phenylethynyl)phthalimide-4-hydroxyphenyl)fluorene (Cardo-HPI) as a diluent into PETIs. Compared to the pristine PETI (PI oligomer), the *T*_g_ of PI/Cardo-40, with Cardo-HPI content increased to 40 wt.%, decreased from 193 °C to 162 °C, indicating that Cardo-HPI functions as a low molecular weight plasticizer. The introduction of Cardo-HPI also elevated the phenylethynyl concentration per unit mass of the blend, prompting an earlier onset of the crosslinking reaction. The minimum melt viscosity of Cardo-HPI was 1.65 Pa·s at 268 °C, lower than that of the PI oligomer, which was 3.8 Pa·s at 306 °C. This reduction was attributed to the low molecular weight and increased free volume induced by the fluorene group. Consequently, the minimum melt viscosity of PI/Cardo-40 decreased to 2.7 Pa·s at 294 °C. The complex viscosity curves are shown in Figure 4C. However, the blend exhibited higher viscosity in the high-temperature region than the PI oligomer, likely due to the earlier onset of crosslinking and the thermal rearrangement of the ortho-hydroxy imide unit into a rigid benzoxazole group. After curing, the *T*_g_, crosslinking density, CTE, and *T*_d5%_ of the PI oligomer were 264 °C, 1.6 × 10^3^ mol/cm^3^, 37.3 ppm/°C, and 459 °C, respectively. By contrast, PI/Cardo-40 exhibited a *T*_g_ of 403 °C, crosslinking density of 27.1 × 10^3^ mol/cm^3^, CTE of 28.1 ppm/°C, and *T*_d5%_ of 470 °C. These improvements were attributed to the uniform dispersion and rigid structure of Cardo-HPI. The TGA curves are shown in Figure 5B and thermal properties of PETI oligomers and cured resins are summarized in Table 3.

Wang et al. [9] reported PETIs based on BPAF, a fluorene-containing dianhydride monomer. The B-O-2 (3,4′-BPDA/4,4′-ODA/PEPA) exhibited a *T*_g_ of 181 °C, while F-O-2 (BPAF/4,4′-ODA/PEPA) showed a higher *T*_g_ of 213 °C, attributed to the more bent and rigid structure of BPAF. F-O-2 was soluble in various organic solvents at room temperature, whereas B-O-2 was only partially soluble upon heating due to the cardo structure of BPAF disrupting the compact chain packing. The minimum melt viscosity of F-O-2 was 74 Pa·s at 339 °C, whereas B-O-2 exhibited a lower value of 35 Pa·s at 328 °C, attributed to the higher molecular weight and rigid, sterically hindered structure of BPAF. The thermal properties of PETI oligomers are summarized in Table 3. And the complex viscosity curves are shown in Figure 4D. After curing, B-O-2 exhibited a *T*_g_ of 383 °C and a *T*_d5%_ of 547 °C, while F-O-2 showed a higher *T*_g_ of 408 °C and *T*_d5%_ of 559 °C, indicating enhanced thermal stability due to the rigidity of BPAF. The TGA curves are shown in Figure 5C and thermal properties of cured resins are summarized in Table 3. BPAF-based polyimides exhibited lower tensile properties compared to previously reported PETI-5 and TriA-PI (tensile strength >100 MPa and elongation at break >13%) due to their high crosslinking density. However, cured F-O-2 demonstrated superior flexural strength of 166 ± 7 MPa compared to 132 ± 5 MPa for cured B-O-2, reflecting better flexural properties owing to the higher chain stiffness of BPAF-based polyimides compared to 3,4′-BPDA-based polyimides.

#### 2.2.3. Asymmetric Structure

**Scheme 4:**
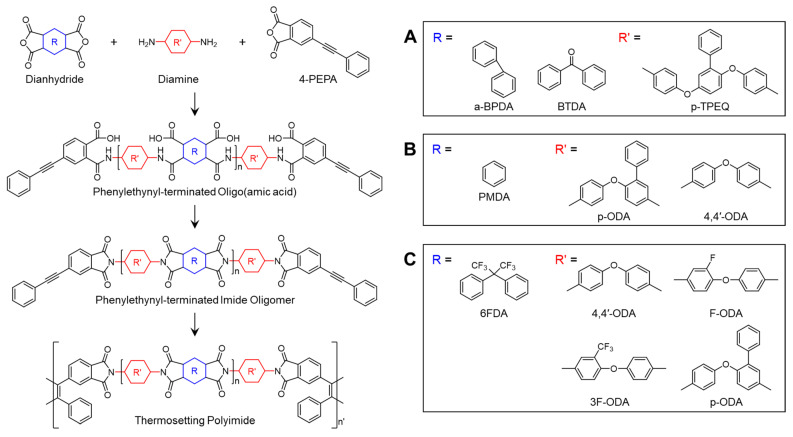
Chemical structures of PETIs with asymmetric configurations: (**A**) Oligo-s–a1–9 and Oligo-bt–a1–9 [48]; (**B**) PMDA/p-ODA/PEPA imide oligomer [10]; (**C**) PETI-H, PETI-F, PETI-3F, and PETI-P [46].

Introducing bulky substituent pendants into monomers can synthesize polymers with asymmetric and non-planar structures, reducing intramolecular and intermolecular packing, decreasing melt viscosity and enhancing solubility [46,47]. Consequently, irregular and bulky substituent monomers can improve the processability of PETIs without significantly compromising backbone rigidity [48]. Studies on PETIs derived from asymmetric monomers are presented. The chemical structures used in the studies presented in this section are shown in Scheme 4.

**Figure 6:**
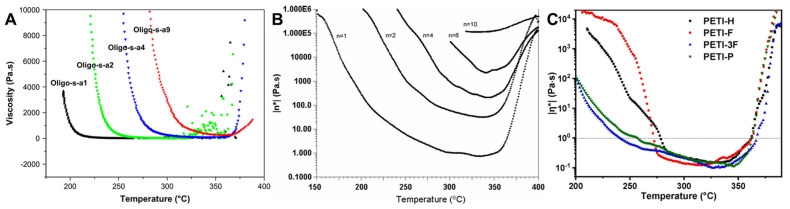
Complex viscosity curves: (**A**) Oligo-s–a1–9 (reprinted with permission from [48], Copyright 2006, Elsevier); (**B**) Uncured PMDA/p-ODA/PEPA imide oligomers (n = 1–10) (reprinted with permission from [10], Copyright 2012, Elsevier); (**C**) PETI-H, PETI-F, PETI-3F, and PETI-P (reprinted with permission from [46], Copyright 2023, Elsevier).

Chen et al. [48] reported PETIs based on the irregular and bulky substituted monomer p-TPEQ. The *T*_g_ of Oligo-s–a1 (s-BPDA/p-TPEQ/PEPA, n = 1) and Oligo-s–a4 (n = 4) were 161.5 °C and 190 °C, respectively. Both PETIs exhibited solubility in DMF at 1 wt.%, while in NMP, Oligo-s–a1 was soluble at 5 wt.% and Oligo-s–a4 at 1 wt.%. The low minimum melt viscosities of Oligo-s–a1 and Oligo-s–a4 were 5 Pa·s at 260 °C and 93 Pa·s at 343 °C, respectively, due to the pendant phenyl groups and diether linkages of p-TPEQ. The complex viscosity curves are shown in Figure 6A. After curing, the *T*_g_ and *T*_d5%_ values were 305 °C and 506 °C for Oligo-s–a1, and 272 °C and 530 °C for Oligo-s–a4. The thermal properties of PETIs are shown in Table 4. Notably, cured Oligo-s–a4 displayed excellent mechanical properties with a strength of 140 MPa, a modulus of 3.4 GPa, and an elongation of 8.8%.

Yokota et al. [10] reported on PETIs based on the asymmetric monomer p-ODA. The PETI derived from PMDA/4,4′-ODA/PEPA was insoluble in NMP and had no observable *T*_g_ below 370 °C, while the PETI derived from PMDA/p-ODA/PEPA (n = 1) exhibited over 33 wt.% solubility in NMP, a *T*_g_ of 152 °C, and a minimum melt viscosity of 1 Pa·s. By contrast, the PMDA/p-ODA/PEPA (n = 4) exhibited similar solubility, with a *T*_g_ of 226 °C and a minimum melt viscosity of 208 Pa·s. The complex viscosity curves are shown in Figure 6B and the thermal properties of PETI oligomers are summarized in Table 4. These findings suggest that PMDA/p-ODA/PEPA-based PETIs possess superior processability compared to conventional PMDA-based PETIs, likely due to the random head-to-tail, head-to-head, and tail-to-tail repeating units that disrupt intermolecular interactions within planar and rigid phenyl-pyromellitimide-phenyl structures. The pendant phenyl group in p-ODA restricts the rotation of the ether bond in 4,4′-ODA and limits both intra- and intermolecular interactions due to its bulky and non-planar structure. The cured resins demonstrated excellent thermal stability, with *T*_g_ and Td5% values of 356 °C and 528 °C for PMDA/p-ODA/PEPA (n = 1) and 346 °C and 539 °C for PMDA/p-ODA/PEPA (n = 4), along with average elongation at break (εb,ave) values of 9.6% and 15.7%, respectively. The thermal properties of cured resins are summarized in Table 4. The high ε_b,ave_ values indicate that the curing reaction mechanism of PEPA was primarily a chain extension rather than cyclization or crosslinking.

**Figure 7:**
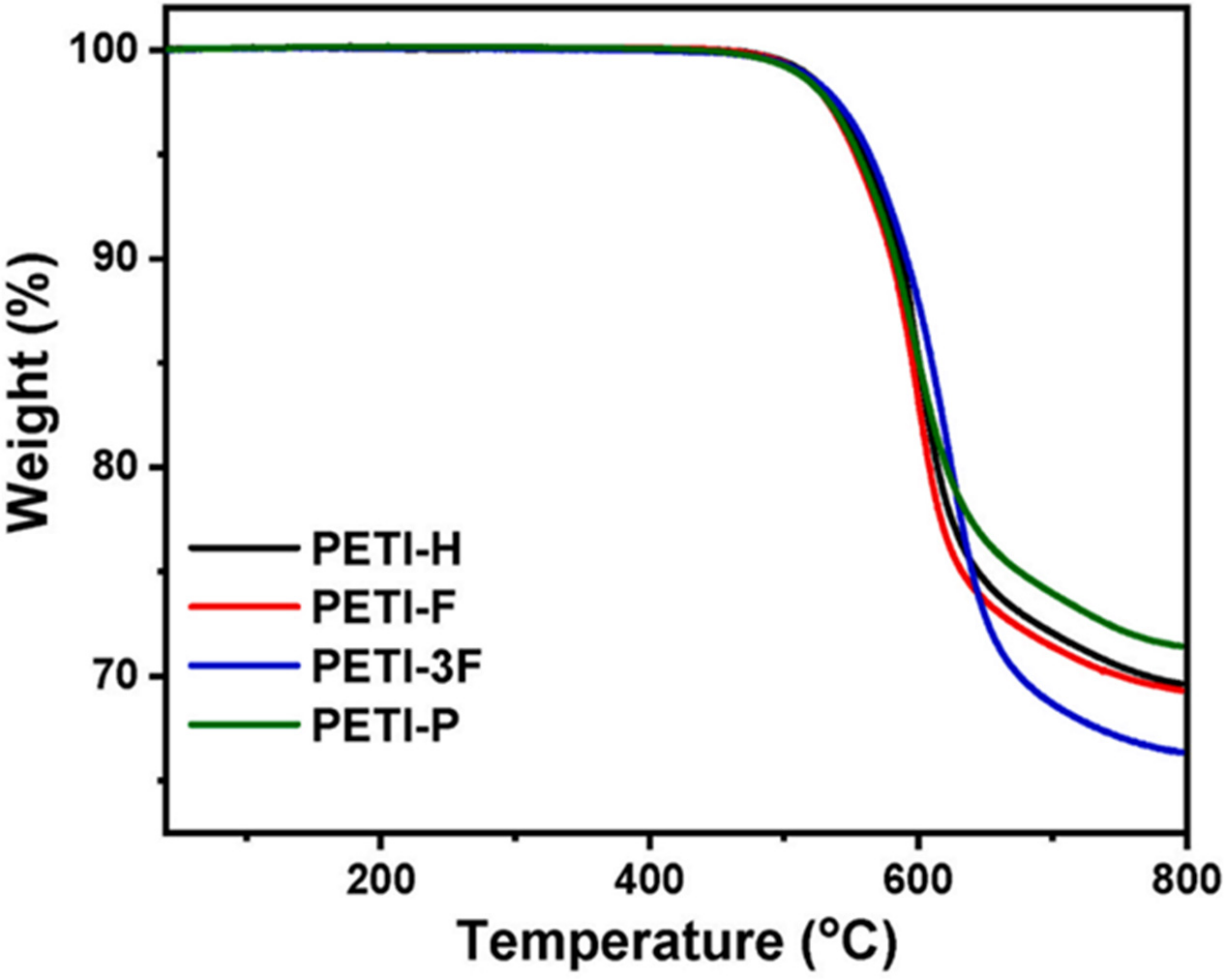
TGA curves: Cured PETI-H, PETI-F, PETI-3F, and PETI-P resins (reprinted with permission from [46], Copyright 2023, Elsevier).

Yang et al. [46] studied PETIs based on 6FDA and asymmetric 4,4′-diaminodiphenyl ether substituted with fluorine (F-ODA), trifluoromethyl group (3F-ODA), or phenyl group (p-ODA), employing an expanded set of diamines. Among these, PETI-P (6FDA/p-ODA/PEPA) did not exhibit melt behavior. The *T*_m_ of PETI-H (6FDA/4,4′-ODA/PEPA), PETI-F (6FDA/F-ODA/PEPA), and PETI-3F (6FDA/3F-ODA/PEPA) decreased in the order PETI-H > PETI-F > PETI-3F. As the volume of the pendant group increased, close packing and intermolecular interactions were reduced, leading to decreased crystallinity and *T*_m_. However, PETI-P exhibited a higher *T*_g_ (140 °C) than PETI-3F (133 °C) due to the larger phenyl group, which caused increased steric hindrance and hindered internal rotation of the backbone. The thermal properties of PETI oligomers and cured resins are summarized in Table 4. PETI-H exhibited partial solubility in most organic solvents due to its symmetric backbone, whereas PETI-3F and PETI-P were soluble in both high-boiling solvents like NMP and low-boiling solvents such as THF and dioxane, attributed to reduced intermolecular interactions and close packing from the introduction of asymmetric units. Consequently, the temperature scale |η*| < 1 Pa·s (T|η*| ≤ 1 Pa·s) was broader for the amorphous forms with lower *T*_m_, with PETI-3F ranging from 241°C to 366 °C and PETI-P from 257 °C to 363 °C, compared to PETI-H (279–361 °C) and PETI-F (275–361 °C), which exhibited lower minimum melt viscosities. The fractional free volume (FFV) of the PETIs increased in the order PETI-P < PETI-H < PETI-F < PETI-3F, influenced by the space-filling effect of the phenyl group within the interstitial chain space. The melt stability of PETI-H, PETI-F, PETI-3F, and PETI-P, evaluated at 280 °C for 4 h, were in the range of 0.36–26.3, 0.20–1.62, 0.36–3.74, and 0.49–323 Pa·s, respectively. The complex viscosity curves are shown in Figure 6C. PETI-H and PETI-P exhibited relatively low melt stability, possibly due to the rigid chains. After curing, PETI-H had the highest *T*_g_ of 463 °C, while PETI-P had the lowest *T*_g_ of 429 °C, likely due to differences in the chain packing. All cured resins demonstrated excellent thermal stability, with *T*_d5%_ values exceeding 553 °C. The TGA curves are shown in Figure 7.

### 2.3. Fluorinated Units in Main Chains

**Scheme 5:**
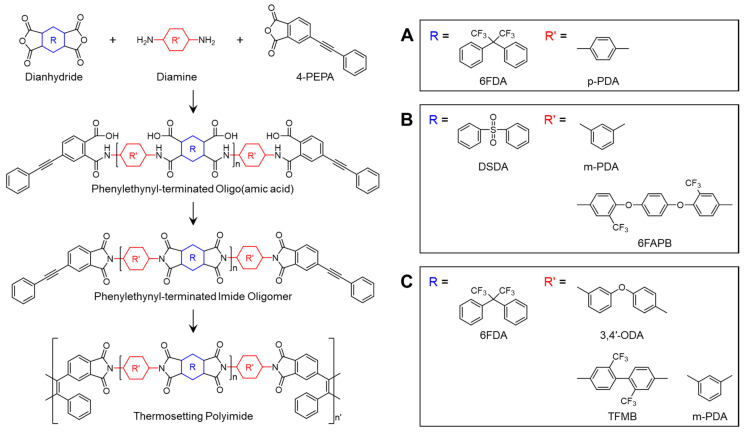
Chemical structures of PETIs with fluorinated units in main chains: (**A**) AFR-PEPA-*N* [16]; (**B**) PI-n [50]; (**C**) PETI-O, PETI-F, and PETI-P [51].

Introducing bulky substituents such as trifluoromethyl groups (–CF_3_) into polymer backbones significantly influences the polymers’ physical properties by increasing packing distance and free volume, which reduces intermolecular interactions and results in lower melt viscosity and improved solubility [14,16,49]. This section presents PETIs with fluorinated units in their main chains. The chemical structures used in the studies presented in this section are shown in Scheme 5.

Morgan et al. [16] reported PETIs based on 6FDA incorporating the (–C(CF_3_)_2_–) group. They synthesized AFR-PEPA-2 (6FDA/p-PDA/PEPA, n = 2) and AFR-PEPA-8 (n = 8), with *T*_g_ values of 194 °C and 260 °C, respectively. AFR-PEPA-2 exhibited a *T*_m_ of 323 °C, whereas AFR-PEPA-8 showed no *T*_m_. Both PETIs displayed excellent thermal stability, with *T*_d5%_ of 566 °C for AFR-PEPA-2 and 557 °C for AFR-PEPA-8, exceeding the *T*_d5%_ values of traditional PETI-5 and 6FDA-modified PETI by over 50 °C. The thermal properties of PETI oligomers and cured resins are summarized in Table 5. Comparatively, PETI-5, with molecular weights of 1250, 2500, and 5000 g/mol, exhibited minimum complex melt viscosities of 5 Pa·s at 335 °C, 90 Pa·s at 335 °C, and 1000 Pa·s at 370 °C, respectively. AFR-PEPA-2, with a calculated number-average molecular weight (*M*_n_) of 1601 g/mol, demonstrated a melt viscosity of 10 Pa·s at 340 °C, while AFR-PEPA-8, with an *M*_n_ of 4699 g/mol, exhibited a melt viscosity of 227 Pa·s at 371 °C, values similar to or lower than those of PETI-5. The reduced melt viscosity of AFR-PEPA-2 and AFR-PEPA-8 was attributed to weaker intermolecular interactions and increased backbone flexibility due to the bulky –CF_3_ groups in the 6FDA structure. Upon curing at 360 °C, AFR-PEPA-2 showed a *T*_g_ of 370 °C, a Young’s modulus of 3.67 GPa, a tensile strength of 50.2 MPa, and a failure strain of 1.5%. When cured at 390 °C, these values improved to a *T*_g_ of 382 °C, a Young’s modulus of 3.35 GPa, a tensile strength of 63.9 MPa, and a failure strain of 2.2%, indicating excellent mechanical properties, likely due to the elimination of network defects from unreacted groups.

**Figure 8:**
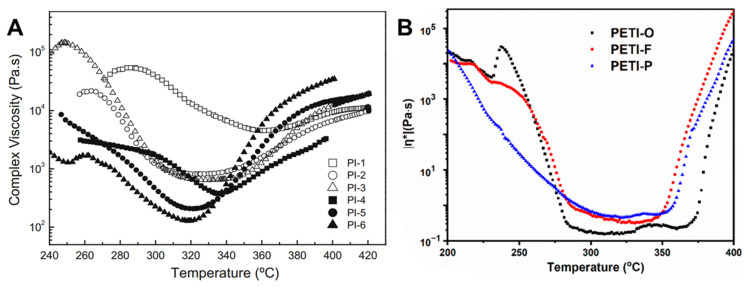
Complex viscosity curves: (**A**) PI-n (reprinted with permission from [50], Copyright 2006, Elsevier); (**B**) PETI-O, PETI-F, and PETI-P (reprinted with permission from [51], Copyright 2021, MDPI).

Fan et al. [50] reported PETIs based on the 6FAPB diamine containing trifluoromethyl groups (–CF_3_). The synthesized PETIs were designated as PI-1 (DSDA/m-PDA/PEPA), PI-2 (DSDA/m-PDA;6FAPB(75:25)/PEPA), and PI-6 (DSDA/6FAPB/PEPA). PI-1 was soluble only in highly polar solvents such as NMP and DMF, while PI-6 exhibited solubility in less polar solvents like DMSO, THF, and even non-boiling solvents such as acetone and chloroform. The minimum melt viscosity of PI-1 was 4545 Pa·s at 361 °C, whereas PI-6, which contains a flexible ether linkage and bulky trifluoromethyl substituents, demonstrated a significantly lower minimum melt viscosity of 128 Pa·s at 318 °C. The complex viscosity curves are shown in Figure 8A. This reduction in viscosity was attributed to the flexible ether bridge structure and the bulky trifluoromethyl groups, which disrupt compact chain packing and reduce polymer density. The *T*_g_ of PI-2 and PI-6 were 207 °C and 199 °C, respectively. Upon curing, PI-2 exhibited a *T*_g_ of 305 °C, as measured by DSC, while the *T*_g_ of cured PI-6 was unobservable. As the mole fraction of 6FAPB increased, the *T*_g_ of the PETIs decreased. The *T*_d5%_ of cured PI-2 and PI-6 were 530 °C and 533 °C, respectively, demonstrating excellent thermal stability. The high thermal stability of 6FAPB, despite its flexible structure, is likely related to the presence of trifluoromethyl groups. The thermal properties of PETI oligomers and cured resins are summarized in Table 5.

**Figure 9:**
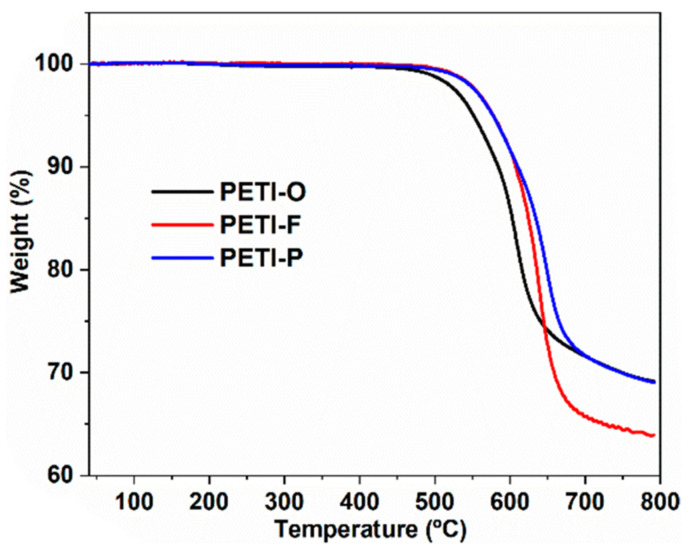
TGA curves: Cured PETI-O, PETI-F, and PETI-P in nitrogen (reprinted with permission from [51], Copyright 2021, MDPI).

Yang et al. [51] reported on PETIs based on 6FDA containing the (–C(CF_3_)_2_–) group, in combination with 3,4′-ODA, m-PDA, and TFDB (substituted with –CF_3_ groups). The *T*_g_ of PETI-O (6FDA/3,4′-ODA/PEPA), PETI-F (6FDA/TFDB/PEPA), and PETI-P (6FDA/m-PDA/PEPA) were 157 °C, 170 °C, and 158 °C, respectively. PETI-F had the highest *T*_g_, likely due to the rigidity and linearity introduced by the –CF_3_ substituted biphenyl structure. PETI-O and PETI-F exhibited clear melting endotherms, whereas PETI-P did not exhibit any melting behavior. The thermal properties of PETI oligomers and cured resins are summarized in Table 5. PETI-F showed unique crystalline properties, with PETI-O displaying clear diffraction peaks and PETI-P exhibiting a broad amorphous peak in wide-angle X-ray diffraction (WXRD) patterns, suggesting that the bent chain structure introduced by m-PDA reduced chain packing and crystallinity. The minimum melt viscosities of PETI-O, PETI-F, and PETI-P were 0.15 Pa·s at 309 °C, 0.31 Pa·s at 331 °C, and 0.45 Pa·s at 323 °C, respectively. The lower melt viscosity of PETI-F compared to PETI-P was attributed to the noncoplanar twisted biphenyl structure and increased free volume due to the bulky –CF_3_ groups. The complex viscosity curves are shown in Figure 8B. Melt stability was assessed by monitoring melt viscosity variation over 2 h at 280 °C, revealing values of 0.52–2.17 Pa·s for PETI-O, 2.41–38.9 Pa·s for PETI-F, and 1.52–19.1 Pa·s for PETI-P. These results indicate that the –CF_3_ group enhanced the electron-withdrawing ability of the PETI backbone, thereby increasing curing reactivity, while the –O– group decreased this ability, leading to higher curing reactivity. The *T*_d5%_ of cured PETI-O, PETI-F, and PETI-P were 558 °C, 578 °C, and 577 °C, respectively, demonstrating excellent thermal stability. The TGA curves are shown in Figure 9. All cured resins also exhibited good mechanical properties, with tensile strengths ranging from 50.8 to 66.2 MPa, tensile moduli from 1.96 to 2.40 GPa, and failure strains from 2.5% to 3.4%.

### 2.4. Flexible Linkages in Main Chains

**Scheme 6:**
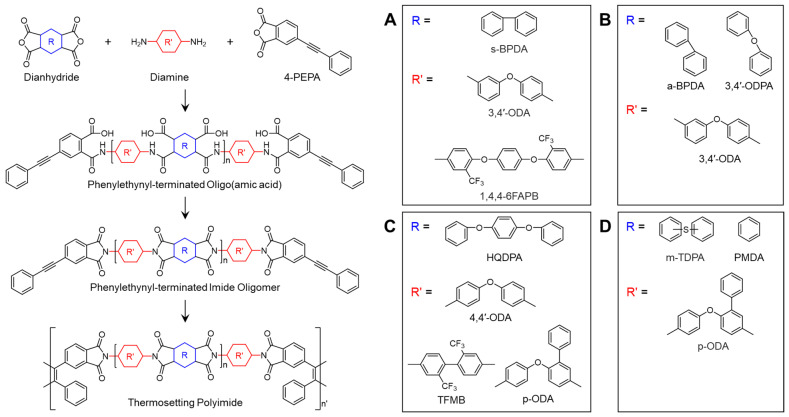
Chemical structures of PETIs with flexible units in main chains: (**A**) PI-n [11]; (**B**) Oligo-n [52]; (**C**) o-O-n, o-T-n, and o-p-n [12]; (**D**) Oligo-n [13].

Aromatic polyimides are generally known for their poor processability due to a rigid polymer backbone and strong intermolecular interactions [11]. The introduction of flexible units, such as ether (–O–) or thioether linkages (–S–), into the polymer backbone imparts flexibility to the polymer chains, which can lower the *T*_m_ and melt viscosity or enhance the solubility of the polyimides, thereby improving their processability [52]. This section reviews several studies on phenylethynyl-terminated imide oligomers incorporating flexible units. The chemical structures used in the studies presented in this section are shown in Scheme 6.

**Figure 10:**
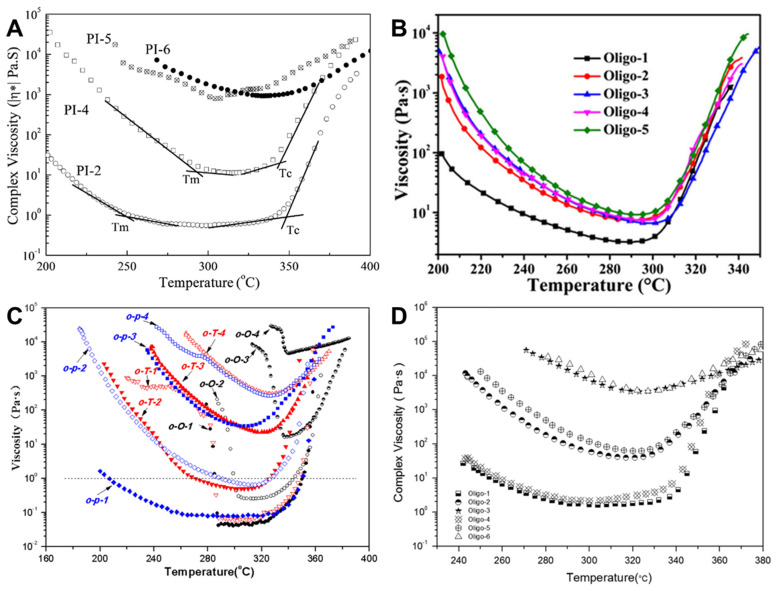
Complex viscosity curves: (**A**) PI-n (reprinted with permission from [11], Copyright 2007, Elsevier); (**B**) Oligo-n (reprinted with permission from [52], Copyright 2018, Springer); (**C**) o-O-n, o-T-n, and o-p-n (reprinted with permission from [12], Copyright 2019, SAGE Publications); (**D**) Oligo-n (reprinted with permission from [13], Copyright 2016, SAGE Publications).

Yang et al. [11] reported a new series of PETIs based on 1,4,4-6FAPB and 3,4′-ODA diamines containing flexible ether groups. The synthesized PETIs were designated as PI-2 (s-BPDA/1,4,4-6FAPB; 3,4′-ODA (50:50)/PEPA, *M*_n_ = 1250), PI-4 (*M*_n_ = 2500), PI-5 (*M*_n_ = 5000), and PI-6 (*M*_n_ = 10,000). The *T*_m_ of PI-2, PI-4, PI-5, and PI-6 were 250 °C, 290 °C, 300 °C, and 325 °C, respectively, with minimum melt viscosities of 0.6 Pa·s (at 299 °C), 11.2 Pa·s (at 315 °C), 750 Pa·s (at 306 °C), and 934 Pa·s (at 332 °C), respectively, indicating that viscosity increases with molecular weight. The complex viscosity curves are shown in Figure 10A. Upon maintaining the temperature at 300 °C for 2 h, the melt viscosity of PI-4 increased from 17.1 to 10,350 Pa·s, while PI-2 exhibited a range from 0.4 to 1391 Pa·s, suggesting that melt stability is closely related to molecular weight and chemical structure. The *T*_g_ of PI-2, PI-4, PI-5, and PI-6 were 124 °C, 180 °C, 209 °C, and 238 °C, respectively, showing a trend similar to the melt viscosity. However, *T*_g_ values measured by DSC and DMA after curing increased as the molecular weight decreased, attributed to higher crosslinking density in lower molecular weight samples. The thermal properties of PETI oligomers and cured resins are summarized in Table 6. The *T*_d5%_ were 570 °C, 578 °C, 603 °C, and 599 °C, respectively, indicating excellent thermal stability. The mechanical properties of the cured polyimides exhibited tensile strengths ranging from 34.5 to 114.3 MPa and elongation at break between 2.5% and 13.8%. As the *M*_n_ increased from 1250 to 5000, both tensile strength and elongation at break improved. However, beyond an *M*_n_ of 5000, no further enhancement in mechanical properties was observed.

**Figure 11:**
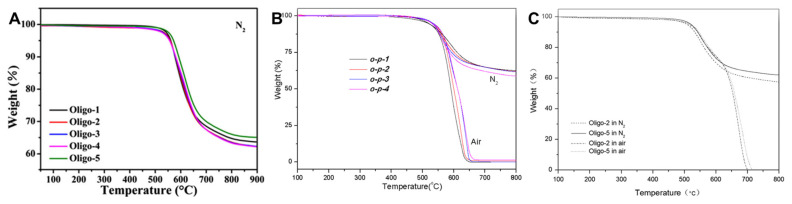
TGA curves: (**A**) Oligo-n (reprinted with permission from [52], Copyright 2018, Springer); (**B**) o-p-n (reprinted with permission from [12], Copyright 2019, SAGE Publications); (**C**) Oligo-2 and Oligo-5 (reprinted with permission from [13], Copyright 2016, SAGE Publications).

Hu et al. [52] reported PETIs based on a-ODPA containing flexible ether units, a-BPDA dianhydrides, and 3,4′-ODA diamine. The synthesized oligomers, designated as Oligo-1 (a-ODPA/3,4′-ODA/PEPA), Oligo-4 (a-ODPA; a-BPDA (20:80)/3,4′-ODA/PEPA), and Oligo-5 (a-BPDA/3,4′-ODA/PEPA), exhibited *T*_g_ of 185 °C, 192 °C, and 201 °C, respectively. After curing, the *T*_g_ values measured by DMA were 317 °C for Oligo-1, 334 °C for Oligo-4, and 338 °C for Oligo-5, attributed to PEPA crosslinking. The *T*_g_ values before and after curing increased with higher a-BPDA content. The thermal properties of PETI oligomers and cured resins are summarized in Table 6. All uncured PETIs were highly soluble in strongly polar solvents such as NMP, DMF, DMAc, and DMSO due to the flexible ether linkages and the asymmetric structure of the monomers within the chains. The minimum melt viscosities of Oligo-1, Oligo-4, and Oligo-5 were reported as 3.2 Pa·s (at 290 °C), 7.4 Pa·s (at 296 °C), and 9.2 Pa·s (at 292 °C), respectively. The complex viscosity curves are shown in Figure 10B. PETIs with a higher a-ODPA content exhibited lower minimum melt viscosities and greater melt viscosity stability, enhancing their processability. The tensile strengths of all cured resins were close to or above 100 MPa, with strains ranging from 10.3% to 13.8%. A comparison of the CTE values for Oligo-4 and Oligo-5 films revealed that Oligo-4 exhibited higher CTE values in both the glassy and rubbery states, attributed to the reduced in-plane orientation due to increased ether linkages in the main chain. The *T*_d5%_ in both nitrogen and oxygen atmospheres were above 556 °C and 558 °C, respectively, indicating excellent thermal stability. The TGA curves are shown in Figure 11A.

Wang et al. [12] reported a new series of PETIs synthesized from HQDPA dianhydride with flexible ether groups and diamines 4,4′-ODA, TFDB, and p-ODA. The PETIs were designated as follows: o-O-1 (HQDPA/4,4′-ODA/PEPA, *M*_n_ = 750), o-T-1 (HQDPA/TFDB/PEPA, *M*_n_ = 750), o-p-1 (HQDPA/p-ODA/PEPA, *M*_n_ = 750), o-p-2 (HQDPA/p-ODA/PEPA, *M*_n_ = 1250), o-p-3 (HQDPA/p-ODA/PEPA, *M*_n_ = 2500), and o-p-4 (HQDPA/p-ODA/PEPA, *M*_n_ = 5000). An XRD analysis indicated that the PETIs in the p-ODA series were amorphous, showing no melting peaks in the DSC. The minimum melt viscosities of o-O-1, o-T-1, and o-p-1 were 0.04 Pa·s at 288 °C, 0.06 Pa·s at 288 °C, and 0.04 Pa·s at 296 °C, respectively. The complex viscosity curves are shown in Figure 10C. The melt viscosity variations at 280 °C over 2 h were 34.8–54.8 Pa·s, 81.9–170 Pa·s, and 0.11–0.39 Pa·s, respectively, suggesting that the asymmetric structure and irregular arrangement, caused by the bulky pendant phenyl groups of p-ODA, reduced intra- and intermolecular interactions. The *T*_g_ of the p-ODA series ranged from 118 °C to 202 °C, increasing with molecular weight. However, the *T*_g_ of the cured PETIs, measured by DSC, decreased with increasing *M*_n_ due to lower crosslinking density at higher molecular weights. The series demonstrated excellent thermal stability, with *T*_d5%_ above 522 °C. The TGA curves are shown in Figure 11B. The *T*_g_ of the cured o-p-1 resin, measured by DMA, was 355 °C, which exceeded those of PETI-298 and PETI-330. The thermal properties of PETI oligomers and cured resins are summarized in Table 6. The p-ODA-based cured resins exhibited superior mechanical properties, with tensile strengths ranging from 51 to 95 MPa, moduli of 2.5 to 2.6 GPa, and elongations between 2% and 8%.

Fang et al. [13] reported the synthesis of PETIs based on m-TDPA, which features a flexible thioether linkage (–S–) and p-ODA, known for its bulky substituents. The PETIs synthesized included Oligo-2 (m-TDPA/p-ODA/PEPA, *M*_n_ = 2500) and Oligo-5 (m-TDPA;PMDA (50:50)/p-ODA/PEPA, *M*_n_ = 2500), both of which exhibited high solubility in organic solvents at a 20 wt.% solid content, not only in polar aprotic solvents but in THF and DMSO. This high solubility was attributed to the flexible thioether linkage in m-TDPA, which lowers the rotational energy barrier of the polymer main chain, along with the asymmetric structures of 3,4′-TDPA and 3,3′-TDPA that disrupt molecular chain regularity, thereby reducing intermolecular interactions and chain packing density. The minimum melt viscosity of Oligo-5 was 61.5 Pa·s at 321 °C, higher than that of Oligo-2, which was 38.4 Pa·s at 319 °C. The complex viscosity curves are shown in Figure 10D. The increase in viscosity with higher PMDA content was due to the symmetry and planarity of PMDA. All synthesized m-TDPA-based PETIs exhibited lower melt viscosities than PMDA/p-ODA and Tri-A PI oligomers, highlighting the chain flexibility imparted by the thioether linkage. The *T*_g_ of Oligo-2 and Oligo-5 were reported as 205 °C and 211 °C, respectively, with cured *T*_g_ values measured by DMA being 302 °C and 320 °C, respectively. The higher *T*_g_ of Oligo-5 was due to the rigid PMDA structure. After curing, the *T*_g_ decreased with increasing *M*_n_, attributed to reduced crosslinking density at higher molecular weights. The thermal properties of PETI oligomers and cured resins are summarized in Table 6. The thermal stability of the cured PETIs was excellent, with *T*_d5%_ of 509 °C for Oligo-2 and 521 °C for Oligo-5. The TGA curves are shown in Figure 11C. Notably, most samples exhibited higher decomposition temperatures in air compared to nitrogen, likely due to differing degradation mechanisms of the thioether linkage. The mechanical properties of Oligo-2 included a modulus of 2.9 GPa, a tensile strength of 52 MPa, and elongation of 2.2%, while Oligo-5 exhibited a modulus of 3.0 GPa, a tensile strength of 77 MPa, and elongation of 3.5%. The introduction of the rigid PMDA structure slightly enhanced the mechanical properties of the cured resins.

### 2.5. Inorganic Hybrid Structure

**Scheme 7:**
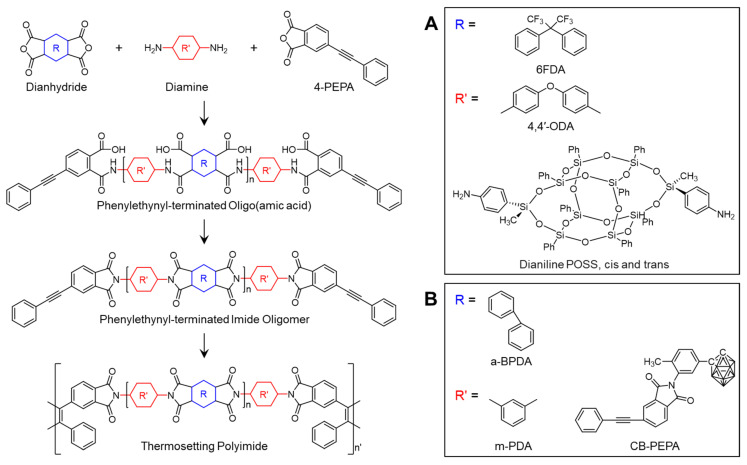
Chemical structures of PETIs with inorganic hybrid configurations: (**A**) 0, 1, 2, 3 cis- and/or trans-POSS (reproduced with permission from [29], Copyright 2013, American Chemical Society); (**B**) PI-mPDA/CB-PEPA blend series (reproduced with permission from [56], Copyright 2017, SAGE Publications).

Polyhedral oligomeric silsesquioxane (POSS) is characterized by a polyhedral structure consisting of a nanoscale inorganic cage made of silicon and oxygen atoms. These hybrid materials combine an inorganic core with an organic periphery, including organic substituents, making them versatile for various applications [53]. Incorporating POSS into materials significantly enhances properties such as wear resistance, thermal stability, resistance to oxidation and ultraviolet radiation, and hydrophobicity [54]. However, the large size of POSS units in copolymers can disrupt the chain packing density and reduce CTC interactions, increasing free volume, which may decrease polymer density and viscoelastic properties [29]. The chemical structures used in the studies presented in this section are shown in Scheme 7.

Conversely, carboranes, which are carbon–boron cluster compounds with a dodecahedral structure (o-, m-, p-C_2_B_10_H_12_), exhibit exceptional thermal stability and hydrophobicity due to their unique cage structure and high boron content [55,56]. When incorporated into a polymer matrix, carborane structures can significantly enhance the thermal stability of the polymer, providing advantages over other boron-containing compounds.

**Figure 12:**
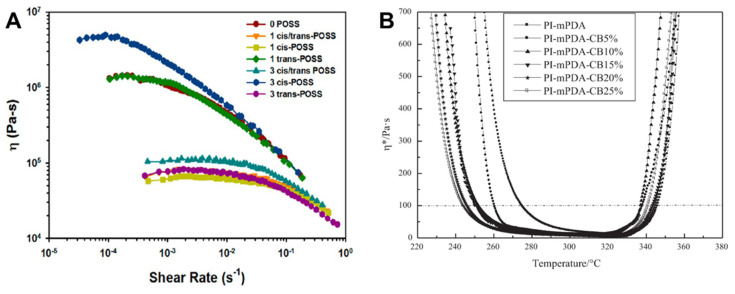
Viscosity curves: (**A**) Steady shear viscosity dependence on shear rate for uncured oligomides at 250 °C (reprinted with permission from [29], Copyright 2013, American Chemical Society); (**B**) PI-mPDA/CB-PEPA blend series (reprinted with permission from [56], Copyright 2017, SAGE Publications).

**Figure 13:**
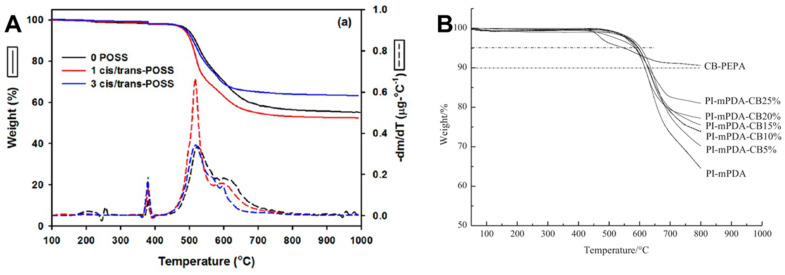
TGA curves: (**A**) Mass loss profiles [solid lines] and their first derivatives [dashed lines] for all oligomers in nitrogen (reprinted with permission from [29], Copyright 2013, American Chemical Society); (**B**) Cured PI-mPDA/CB-PEPA blend series in nitrogen (reprinted with permission from [56], Copyright 2017, SAGE Publications).

Yandek et al. [29] reported on a series of polyimides based on bis(4-anilinylmethylsilyloxy)octaphenylsilsesquioxane (dianiline POSS), including both cis and trans isomers of POSS dianiline. The synthesized PETIs were designated as 0 POSS (6FDA/4,4′-ODA/PEPA), 1 cis- and/or trans-POSS (6FDA/4,4′-ODA;cis- and/or trans-POSS(80:20)/PEPA), and 3 cis- and/or trans-POSS (6FDA/4,4′-ODA;cis- and/or trans-POSS(60:40)/PEPA). When comparing the viscosity of these PETIs at 250 °C under varying shear rates, it was found that most POSS-incorporated PETIs exhibited significantly lower zero-shear viscosity (η_0_) compared to 0 POSS, attributed to the large POSS molecules disrupting compact chain packing and charge transfer complex (CTC) interactions. CTC interactions are characteristic of imides and occur between the electron-rich nitrogen atoms (donors) and the electron-deficient carbonyl groups (acceptors), occasionally behaving as physical cross-links. Thus, as chain packing and CTC interactions decrease, the melt viscosity decreases. However, 1 trans-POSS exhibited behavior similar to 0 POSS, and 3 cis-POSS had the highest η_0_, indicating that the POSS isomer type influenced the packing and orientation of the PETI chains, thereby affecting their response to shear stress. The complex viscosity curves are shown in Figure 12A. After curing, 1 POSS demonstrated 28–38% less water absorption than 0 POSS, and 3 POSS showed 67–70% lower saturated water absorption, indicating superior moisture resistance. Consequently, the *T*_g_ of the saturated 3 cis/trans-POSS polyimide was 10 °C higher than that of 0 POSS. In terms of thermal stability, 0 POSS had the highest *T*_d5%_ at 508 °C, while POSS incorporation decreased thermal stability by 10–15 °C. Despite this, the 3 POSS polyimide exhibited the highest char yield due to the increased concentration of reactive intermediates from phenyl hydrogen during pyrolysis, promoting additional crosslinking. Moreover, in air, the POSS cages oxidized to form silicon dioxide (SiO_2_), which helped suppress mass loss during the initial stages of decomposition. The TGA curves are shown in Figure 13A and the *T*_d5%_ values of cured resins are summarized in Table 7.

Chen et al. [56] investigated the effects of blending PETIs with an imide compound diluent (CB-PEPA) containing the icosahedral carborane, 1-(3-amino-4-tolyl)-1,2-dicarba-dodecaborane (CBNH). The base PETI, PETI-mPDA (a-BPDA/m-PDA/PEPA), exhibited a minimum melt viscosity of 12.2 Pa·s at 325 °C and maintained a melt viscosity below 100 Pa·s within the temperature range of 275–346 °C. By contrast, PI-mPDA-CB25%, with 20 wt.% CB-PEPA added showed a lower minimum melt viscosity of 6.7 Pa·s at 317 °C and a broader processing window, maintaining a melt viscosity below 100 Pa·s over the range of 243–342 °C. The complex viscosity curves are shown in Figure 12B. The cured resins of PI-mPDA and PI-mPDA-CB25% exhibited *T*_d5%_ values of 585 °C and 586 °C, respectively, with char yields of 64.5% and 81.0% at 800 °C, attributed to the incorporation of the carborane cage. Notably, adding 5 wt.% CB-PEPA increased the *T*_d5%_ to 604 °C, although further increases in CB-PEPA content led to a decrease in *T*_d5%_, likely due to the rapid weight loss of CB-PEPA observed around 480 °C. The TGA curves are shown in Figure 13B and *T*_d5%_ values of cured resins are summarized in Table 7.

### 2.6. Liquid Crystalline Mesogenic Structure

**Scheme 8:**
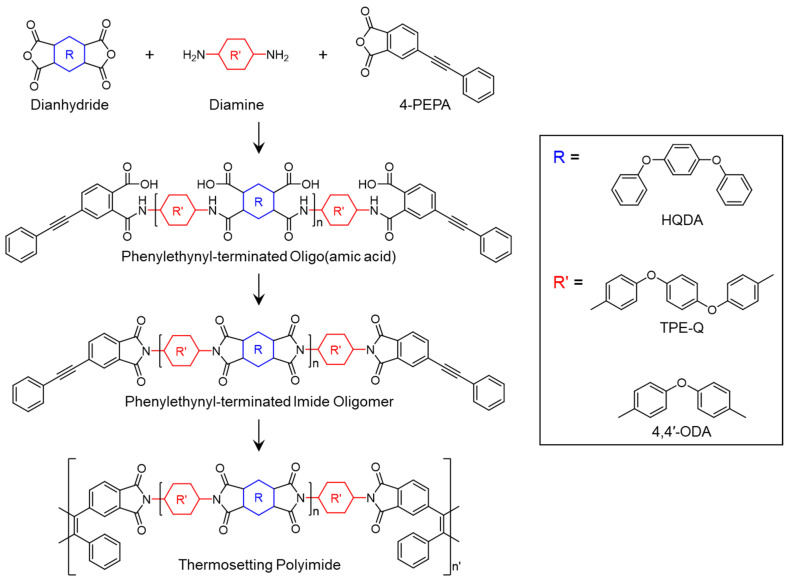
Chemical structures of PETIs with liquid crystalline mesogenic configurations: LC-PI series (reprinted with permission from [58], Copyright 2021, American Chemical Society).

Liquid crystalline polymers (LCPs) exhibit superior mechanical and thermal properties due to the alignment of polymer chains driven by intermolecular interactions [57]. By optimizing the curing temperature within the liquid crystalline behavior range, PETIs can be developed to retain liquid crystalline textures at room temperature, enhancing intermolecular interactions through aligned polymer chains. This alignment can improve heat resistance, thermal conductivity, and mechanical properties. However, the application of this approach to imide oligomers has rarely been explored. Herein, we introduce a PETI that leverages liquid crystallinity for improved performance. The chemical structures used in the study presented in this section are shown in Scheme 8.

**Figure 14:**
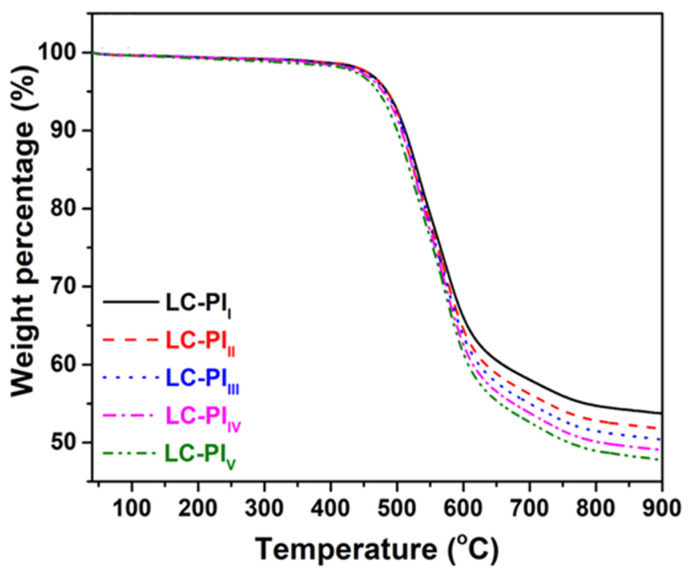
TGA curves: LC-PI series (reprinted with permission from [58], Copyright 2021, American Chemical Society).

Gu et al. [58] reported a series of phenylethynyl-terminated liquid crystalline polyimides (LC-PIs) that exhibit liquid crystalline behavior. The synthesized LC-PIs were designated as LC-PI_I_ (HQDA/4,4′-ODA/PEPA), LC-PI_II_ (HQDA/4,4′-ODA;TPE-Q(75:25)/PEPA), LC-PI_III_ (HQDA/4,4′-ODA;TPE-Q(50:50)/PEPA), LC-PI_IV_ (HQDA/4,4′-ODA;TPE-Q(25:75)/PEPA), and LC-PI_V_ (HQDA/TPE-Q/PEPA). A DSC analysis of the precursor preLC-PI_IV_ showed endothermic peaks at 272 °C and 388 °C, indicating the liquid crystalline range. Within this temperature range, bright yellow dots were observed under POM. XRD spectra displayed a strong peak at 18°, confirming that the liquid crystalline state persisted at room temperature due to crosslinking of the phenylethynyl groups. The cured LC-PI_IV_ resin exhibited the best mechanical properties, with a tensile strength of 119.0 MPa, an elongation at break of 50.3%, a modulus of 2.1 GPa, and a toughness of 55.4 MJ/m^3^. These superior properties were attributed to the microstructural alignment of polymer chains within the liquid crystalline range and the close packing of the chains. Due to the closely packed structure, where molecular chains maintain a microscopically ordered arrangement, the phthalimide mesomorphic units present in the main chain exhibit strong interactions with each other, thereby reinforcing intermolecular interactions between the molecular chains. This promotes stress resistance and enhances the thermal and mechanical properties. The *T*_g_ of LC-PI_IV_ was 329.3 °C, and the *T*_d5%_ was 478.7 °C, demonstrating excellent thermal stability. The TGA curves are shown in Figure 14 and the thermal properties of PETI oligomers and cured resins are summarized in Table 8.

## 3. Conclusions and Perspectives

This review has highlighted significant advancements in the development of ultra-high-temperature PETI oligomers, emphasizing the complex interactions between their molecular structures, processing conditions, and resulting material properties. The incorporation of monomers with noncoplanar configurations, such as kink, and cardo structures, alongside the inclusion of fluorinated groups, flexible linkages, and liquid crystalline mesogenic structures, has enabled the tailoring of PETI properties to meet the demanding requirements of advanced applications. These structural modifications have enhanced the thermal stability, glass transition temperatures, and solvent resistance of PETIs while optimizing processing characteristics, including reduced melt viscosity and improved processability.

Despite these advancements, several challenges remain. The synthesis of PETIs with precisely controlled molecular weights and narrow polydispersity indices remains complex, necessitating further refinement of polymerization techniques. Additionally, the relationship between molecular architecture and macroscopic properties, particularly for ultra-high-temperature applications, requires deeper investigation. Developing predictive models that accurately correlate molecular structure with performance metrics is crucial for further progress.

Future research should focus on exploring novel monomeric structures and polymerization strategies to enhance the thermal, mechanical, and electrical properties of PETIs. Expanding the scope of applications by integrating PETIs into multifunctional composites and hybrid materials could unlock new potentials, particularly in aerospace, electronics, and high-performance coatings. Moreover, addressing the environmental impact and sustainability of PETI production and processing is essential to align this research with global trends toward greener and more sustainable material solutions. By addressing these challenges, the field of PETIs is poised to significantly contribute to the next generation of high-performance materials.

## Figures and Tables

**Table 1:** Thermal properties of PETIs with isomeric structures.

PETI	PETI Oligomer				Cured Resin		
	Calc’d. *M*_n_ (g/mol)	*T*_g_ (°C)	*T*_m_ (°C)	Minimum Melt Viscosity (Pa·s)	*T*_g_ (°C)		*T*_d5%_ (°C) (N_2_)
					DSC	DMA	
Oligo-1.5 [3]	1340	161	–	2000 at 300 °C	341	–	–
Oligo-10 [3]	5240	237	–	200,000 at 365 °C	308	–	–
PI-6 [8]	900	102	251	0.08 at 295 °C	304	352	545
PI-7 [8]	900	110	–	0.11 at 312 °C	292	356	542
Oligo-5 [33]	3388	239	–	120 at 354 °C	335	373	539
Oligo-PMDA [33]	3388	–	–	–	–	–	–
P4 [34]	750	139	–	–	298	–	–
P5 [34]	750	–	268	2300 at 340 °C	–	–	–
PETI-RTM [34]	750	132	–	–	258	–	–

**Table 2:** Thermal properties of PETIs with kink structures.

PETI	PETI Oligomer				Cured Resin		
	Calc’d. *M*_n_ (g/mol)	*T*_g_ (°C)	*T*_m_ (°C)	Minimum Melt Viscosity (Pa·s)	*T*_g_ (°C)		*T*_d5%_ (°C) (N_2_)
					DSC	DMA	
PE-3F-PETI-5K [14]	5000	218	–	59 at 344 °C	272	–	534
PETI-5K [14]	5000	225	349	2237 at 354 °C	270	–	532

**Table 3:** Thermal properties of PETIs with cardo structures.

PETI	PETI Oligomer				Cured Resin		
	Calc’d. *M*_n_ (g/mol)	*T*_g_ (°C)	*T*_m_ (°C)	Minimum Melt Viscosity (Pa·s)	*T*_g_ (°C)		*T*_d5%_ (°C) (N_2_)
					DSC	DMA	
*o*-BAFL-0 [44]	–	218	–	81 at 344 °C	340	–	556 ^a^
*o*-BAFL-25 [44]	–	273	–	1810 at 349 °C	362	–	561 ^a^
Oligo-0 [45]	2500	190	–	24.1 at 334 °C	288	304	523
Oligo-25 [45]	2500	205	–	34.7 at 331 °C	296	322	497
PI oligomer [43]	–	193	–	3.8 at 306 °C	–	264	459
PI/Cardo-40 [43]	–	162	–	2.7 at 294 °C	–	403	470
F-O-2 [9]	1906	213	–	74 at 339 °C	–	408	559
B-O-2 [9]	1578	181	263	35 at 328 °C	–	383	547

^a^ In argon atmosphere.

**Table 4:** Thermal properties of PETIs with asymmetric structures.

PETI	PETI Oligomer				Cured Resin		
	Calc’d. *M*_n_ (g/mol)	*T*_g_ (°C)	*T*_m_ (°C)	Minimum Melt Viscosity (Pa·s)	*T*_g_ (°C)		*T*_d5%_ (°C) (N_2_)
					DSC	DMA	
Oligo-s–a1 [48]	1400	161.5	–	5 at 260 °C	300	305	506
Oligo-s–a4 [48]	3300	190	–	93 at 343 °C	277	272	530
PMDA/p-ODA/PEPA (n = 1) [10]	1190	152	–	1	–	356	528
PMDA/p-ODA/PEPA (n = 4) [10]	2570	226	–	208	–	346	539
PETI-H [46]	1000	–	172,276	0.14 at 321 °C	–	463	559
PETI-F [46]	1000	–	161,269	0.12 at 313 °C	–	454	553
PETI-3F [46]	1000	133	200	0.098 at 326 °C	–	452	564
PETI-P [46]	1000	140	–	0.11 at 345 °C	–	429	555

**Table 5:** Thermal properties of PETIs with fluorinated units in main chains.

PETI	PETI Oligomer				Cured Resin		
	Calc’d. *M*_n_ (g/mol)	*T*_g_ (°C)	*T*_m_ (°C)	Minimum Melt Viscosity (Pa·s)	*T*_g_ (°C)		*T*_d5%_ (°C) (N_2_)
					DSC	DMA	
AFR-PEPA-2 [16]	1601	194	323	1 at 371 °C	370	–	568
AFR-PEPA-8 [16]	4699	260	–	54 at 410 °C	358	–	557
PI-2 [50]	5000	207	–	377 at 336 °C	305	323	530
PI-6 [50]	5000	199	–	128 at 318 °C	–	278	533
PETI-O [51]	1000	157	215, 246	0.15 at 309 °C	–	363	558
PETI-F [51]	1000	170	214, 273	0.31 at 331 °C	–	438	578
PETI-P [51]	1000	158	–	0.45 at 323 °C	–	398	577

**Table 6:** Thermal properties of PETIs with flexible units in main chains.

PETI	PETI Oligomer				Cured Resin		
	Calc’d. *M*_n_ (g/mol)	*T*_g_ (°C)	*T*_m_ (°C)	Minimum Melt Viscosity (Pa·s)	*T*_g_ (°C)		*T*_d5%_ (°C) (N_2_)
					DSC	DMA	
PI-2 [11]	1250	124	250	0.6 at 299 °C	330.9	354	570
PI-4 [11]	2500	180	290	11.2 at 315 °C	289.8	316	578
PI-5 [11]	5000	209	300	750 at 306 °C	281.2	310	603
PI-6 [11]	10,000	238	325	934 at 332 °C	276.3	314	599
Oligo-1 [52]	2543	185	–	3.2 at 290 °C	285	317	559
Oligo-4 [52]	2505	192	–	7.4 at 296 °C	305	334	556
Oligo-5 [52]	2493	201	–	9.2 at 292 °C	311	338	572
o-O-1 [12]	750	–	290	0.04 at 288 °C	–	–	526
o-T-1 [12]	750	154	287	0.06 at 288 °C	289	–	541
o-p-1 [12]	750	118	–	0.04 at 296 °C	–	355	522
o-p-2 [12]	1250	156	–	0.64 at 314 °C	280	307	534
o-p-3 [12]	2500	188	–	33.4 at 308 °C	262	261	533
o-p-4 [12]	5000	202	–	0.08 at 270 °C	243	241	531
Oligo-2 [13]	2500	205	–	38.4 at 319 °C	294	302	509
Oligo-5 [13]	2500	211	–	61.5 at 321 °C	305	320	521

**Table 7:** Thermal properties of PETIs with inorganic hybrid structures.

PETI	PETI Oligomer		Cured Resin
	Calc’d. *M*_n_ (g/mol)	Minimum Melt Viscosity (Pa·s)	*T*_d5%_ (°C) (N_2_)
0 POSS [29]	3094	–	508
1 cis/trans-POSS [29]	4230	–	499
1 cis-POSS [29]	4230	–	493
1 trans-POSS [29]	4230	–	494
3 cis/trans-POSS [29]	6501	–	499
3 cis-POSS [29]	6501	–	493
3 trans-POSS [29]	6501	–	493
PI-mPDA [56]	–	12.2 at 325 °C	585
PI-mPDA-CB5% [56]	–	11.8 at 305 °C	604
PI-mPDA-CB25% [56]	–	6.7 at 317 °C	586

**Table 8:** Thermal properties of PETIs with liquid crystalline mesogenic structures.

PETI	PETI Oligomer		Cured Resin	
	Calc’d. *M*_n_ (g/mol)	*T*_m_ (°C)	*T*_g_ (°C)	*T*_d5%_ (°C) (N_2_)
			DSC	
LC-PI_III_ [58]	20,800	353	330.2	482.2
LC-PI_IV_ [58]	21,600	272	329.3	478.7
LC-PI_V_ [58]	22,300	213	327.2	471.2
